# Trends in cannabis consumption: psychotic and anxiety symptoms among users

**DOI:** 10.1192/j.eurpsy.2024.247

**Published:** 2024-08-27

**Authors:** O. Martin-Santiago, P. Martinez.Gimeno, M. Calvo-Valcarcel, C. Alario-Ruiz, B. Arribas-Simon

**Affiliations:** Hospital Clinico Universitario, Valladolid, Spain

## Abstract

**Introduction:**

During cannabis use, some individuals may experience psychotic symptoms, such as unusual perceptions or irrational thoughts, including mild hallucinations or temporary paranoia. Anxiety is also common, characterized by excessive worry or intense fear. The occurrence of these symptoms varies based on cannabis quantity, individual sensitivity, and surroundings. Although not all users experience these effects, the link between cannabis and psychotic or anxiety symptoms highlights the need for a thorough risk assessment.

**Objectives:**

Our goal is to analyze trends in cannabis use, as well as the psychotic and anxiety symptoms experienced by users, and to examine whether cannabis use is associated with other substances consumption.

**Methods:**

We collected demographic and substance consumption data from two groups: 29 individuals aged 18 to 28 who had tried cannabis at least once and 19 regular consumers through a structured questionnaire.

**Results:**

Regular cannabis consumers had a higher proportion of males than those who had tried it once (*X²*_(1)_=4.81; p=0.028). There were no significant differences in age, alcohol or tobacco consumption between the groups. Notably, regular cannabis consumers had a history of using other illegal drugs, both in the past and within the last month (*X²*_(1)_=8.53; p=0.003). Regarding cannabis effects, regular users more frequently reported sensations like euphoria, relaxation, altered time perception, tachycardia, motor coordination difficulties, and impaired clear thinking compared to one-time users (X²_(1)_=10.12; p=0.001). Regarding anxiety symptoms during cannabis consumption, both groups experienced a similar frequency. Finally, regular cannabis consumers reported strange ideas or perceptions more often than one-time users (*X²*_(1)_=0.743; p=0.019). However, the associated discomfort level was similar in both groups.

**Conclusions:**

This study highlights that regular cannabis use is associated with a greater likelihood of using other substances and experiencing more pronounced effects, including psychotic symptoms. However, it doesn’t necessarily lead to increased anxiety symptoms compared to one-time users. It’s important to acknowledge that the relationship between cannabis and psychosis is intricate and influenced by factors like consumption quantity and individual sensitivity. These findings stress the importance of understanding cannabis’s impact on mental health and its connection to the use of other substances.

**Disclosure of Interest:**

None Declared

